# Loss of plexin-B3 in hepatocellular carcinoma

**DOI:** 10.3892/etm.2015.2243

**Published:** 2015-01-30

**Authors:** YUWU LIU, CHANG WU, YING WANG, SAILAN WEN, JUNPU WANG, ZHIHONG CHEN, QIONGQIONG HE, DEYUN FENG

**Affiliations:** 1 Department of Pathology, Xiangya Hospital, Central South University, Changsha, Hunan 410008, P.R. China; 2 Department of Pathology, School of Basic Medical Sciences, Central South University, Changsha, Hunan 410013, P.R. China; 3 Department of Morphology, The Institute of Advanced Occupation Technology, Xinjiang Medical University, Ürümqi, Xinjiang 830011, P.R. China; 4 Department of Pathology, Shenzhen Sixth People’s Hospital (Nanshan Hospital), Shenzhen, Guangdong 518052, P.R. China

**Keywords:** plexin-B3, hepatocellular carcinoma

## Abstract

Plexins are the primary receptors of semaphorins, and participate in the majority of intracellular pathways triggered by semaphorins, including the regulation of cell adhesion and the motility of numerous cell types. Recently, several studies have reported that plexins can significantly affect different aspects of cancer cell biology, and the aberrant expression of plexins has been observed in a wide variety of tumor types. However, the expression and role of plexin-B3 in hepatocellular carcinoma (HCC) is yet to be investigated. In the present study, plexin-B3 expression was measured in 14 paired HCC samples and the corresponding adjacent non-cancerous tissue by quantitative polymerase chain reaction and western blot analysis. The results indicated that the mRNA and protein expression levels of plexin-B3 were downregulated in HCC samples when compared with the corresponding adjacent non-cancerous tissue. In order to elucidate the correlation between clinicopathological data and the expression of plexin-B3 in patients with HCC, 84 HCC archived specimens were analyzed by immunohistochemistry (IHC). The IHC results revealed that the protein expression level of plexin-B3 was lower in the HCC samples compared with the corresponding adjacent non-cancerous tissue, and plexin-B3 underexpression was correlated with the patient gender and tumor size. In conclusion, these results indicated that loss of plexin-B3 in HCC may be of predictive value for the occurrence and progression of HCC. Thus, plexin-B3 may be a promising biomarker for the diagnosis and treatment of tumors in the future.

## Introduction

Liver cancer remains the fifth most common malignancy in males and the seventh in females worldwide. An estimate indicated that 748,300 new liver cancer cases and 695,900 cancer mortalities occurred worldwide in 2008 (1). HCC is the major histological subtype among primary liver cancer, accounting for 70–85% of all primary liver cancer cases (2). Although there have been advances in the diagnosis and treatment of HCC in recent years, the prognosis for patients with HCC remains poor (3). Diagnosis during the later stages of HCC, with the development of clinical symptoms and a high rate of recurrence or metastases following curative resection, are considered the main reasons for poor prognosis (4,5). Thus, further investigation into the mechanisms underlying HCC may provide a reliable scientific basis for improving treatment methods and diagnosis.

Semaphorins comprise a large family of secreted or membrane-bound proteins that function as crucial regulators of morphogenesis and homeostasis in a wide range of organ systems, subsequently influencing a variety of biological processes from cell migration to cytokine release (6–8). In addition, semaphorins regulate tumor angiogenesis, tumor growth, cancer cell invasiveness and metastatic spreading (9). The cellular functions of semaphorins are the result of the receptors, plexins and neuropilins (10). Plexins can be divided into four homology groups, named the plexin-A (plexin-A1, plexin-A2, plexin-A3 and plexin-A4), -B (plexin-B1, plexin-B2 and plexin-B3), -C (plexin-C1) and -D (plexin-D1) subfamilies (10).

Plexin-B3 plays an important role as a regulator in a multitude of biological processes and the occurrence of tumors (11,12). The functions of plexin-B3 are associated with semaphorin 5A (Sema5A), as a high-affinity receptor (13,14). Upon semaphorin-independent signaling mechanisms, plexin-B3 influences neuronal morphogenesis or function and interacts with Rin (15). Li *et al* reported that plexin-B3, upon stimulation by its ligand Sema5A, can inhibit the migration and invasion of glioma cells (16). Plexin-B3 mutations have been identified in prostate cancer (17), breast cancer (18) and melanoma (19). In addition, the overexpression of Sema5A and plexin-B3 in gastric carcinoma have been shown to correlate with the invasion and metastasis of tumors (20). However, the expression and localization of plexin-B3 in HCC remain unknown.

In the present study, the mRNA and protein expression levels of plexin-B3 were analyzed in HCC samples and corresponding adjacent non-cancerous tissue, in order to preliminarily analyze the association with the occurrence of HCC.

## Materials and methods

### Samples and clinicopathological data

Paired HCC samples and the corresponding adjacent non-cancerous tissues were obtained from 14 patients who had undergone a liver resection at the Xiangya Hospital of Central South University (Changsha, China). The tissue samples were immediately snap-frozen in liquid nitrogen, and stored long-term at −80°C in a freezer. Of the 14 patients, 12 were male and two were female, with a gender ratio of 6:1 (male/female). The mean age of the patients was 52 years old.

In total, 84 HCC archived specimens were obtained from the tissue bank of the Department of Pathology in the Xiangya Hospital of Central South University. All patients had been treated surgically between 2011 and 2012, and the HCC specimens had been routinely processed with 10% formalin fixation and paraffin embedding prior to archiving. The clinical and pathological features of the 84 HCC cases were described briefly. Of the 84 patients, 68 were male and 16 were female, with a gender ratio (male/female) of 4.25:1. The mean age of the patients was 50 years. According to the microscopic pathological characteristics, the histological grade of tumor differentiation was assigned. In total, 14% of tumors were well-differentiated, 64% were moderately-differentiated and 21% of tumors were classified with poor differentiation. The majority of patients (70 cases) had a single tumor, while 11 cases had multiple tumors. A microscopic capsule and/or vascular invasion was observed in ~69% of the patients. Tumor staging was conducted and 36 cases were assigned as stage I, 40 cases were stage II, seven cases were stage III and one case was classified as stage IV. Hepatic cirrhosis was recorded in 58% of the patients. All pathological diagnoses were based on the World Health Organization’s criteria (21), while histological classification and tumor differentiation were conducted according to the Edmondson and Steiner grading system (22). Tumor staging was defined according to to the Sixth Edition of TNM Classification guidelines, which was jointly promulgated by the American Joint Committee on Cancer and the International Union Against Cancer (23).

Written informed consent was provided by all the patients, and all the experimental protocols of the study were approved by the Ethics Committee of Xiangya Hospital of Central South University.

### Quantitative polymerase chain reaction (PCR)

Total RNA was extracted from the frozen tumor specimens using TRIzol reagent (Invitrogen Life Technologies, Carlsbad, CA, USA). A 2-μg sample of total RNA was used for the synthesis of Oligo (dT)-primed single stranded cDNA using a First Strand cDNA Synthesis kit (Fermentas, Burlington, ON, Canada). The cDNA products were amplified using a SYBR PrimeScript RT-PCR kit (Takara Bio, Inc., Otsu, Japan), according to the manufacturer’s instructions. GAPDH served as an internal control for the total cDNA content. The sequence of oligonucleotides used as PCR primers were as follows: Plexin-B3 forward, 5′-GGCTGGTCACCTGACCCTAT-3′ and reverse, 5′-CCCACTGTTGCTCCATCTG-3′; GAPDH forward, 5′-AGGCTAGCTGGCCCGATTTC-3′ and reverse, 5′-TGGCAACAATATCCACTTTACCAGA-3′. The relative mRNA expression levels of plexin-B3 were measured using Ct values, corrected for GAPDH expression, according to the following equation: 2^−ΔCt^ [ΔCt = Ct (target gene) − Ct (internal control)]. All experiments were performed in triplicate.

### Western blot analysis

Frozen tumor specimens were treated in radioimmunoprecipitation assay lysis buffer (50 mM Tris-HCl, 150 mM NaCl, 1% Triton X-100, 1% sodium deoxycholate and 0.1% SDS) containing a protease inhibitor cocktail (Roche Diagnostics, Basel, Switzerland) for 30 min on ice. Equal amounts of protein were separated by 10% SDS-PAGE, and transferred onto polyvinylidene difluoride membranes (Sigma-Aldrich Shanghai Trading Co., Ltd., Shanghai, China). After blocking with 5% skim milk solution for 2 h, the membranes were incubated with a rabbit polyclonal plexin-B3 antibody (1:200; cat. no. sc-67144, Santa Cruz Biotechnology, Inc., Santa Cruz, CA, USA) or a mouse monoclonal β-actin antibody (1:1,000; cat. no. 60008-1-Ig, Proteintech, Chicago, IL, USA) at 4°C overnight. The membranes were then incubated with appropriate secondary antibodies: Goat anti-rabbit (cat. no. sc-2004) or goat anti-mouse (cat. no. sc-2005) horseradish peroxidase (HRP)-conjugated immunoglobulin G (1:2,000; Santa Cruz Biotechnology, Inc.) for 1 h at room temperature, and the signal of the protein was revealed using an enhanced chemiluminescence method (Auragene Bioscience, Inc., Changsha, China). Band intensities were quantified using image analysis software (Bio-Rad Laboratories, Hercules, CA, USA). Each experiment was repeated a minimum of three times.

### Immunohistochemistry (IHC)

IHC analysis of the paraffin-embedded sections was performed according to a two-step protocol (Polink-2 Plus Polymer HRP Detection System; Golden Bridge International, Inc., Bothell, WA, USA). Briefly, the sections were deparaffinized in xylene and hydrated in a graded series of ethanol (100–50%) and tap water. A high pressure method was selected to perform antigen retrieval in citrate buffer (0.01 M, pH 6.0). Subsequently, the sections were incubated in 3% H_2_O_2_ at room temperature for 10 min to block the endogenous peroxidase activity. After washing in phosphate-buffed saline, the sections were treated with an anti-plexin-B3 antibody (Santa Cruz Biotechnology, Inc.) at 4°C overnight. The sections were incubated with a polymer helper (Golden Bridge International, Inc.) at 37°C for 20 min, followed by incubation with a poly-horseradish peroxidase-conjugated anti-mouse/rabbit IgG at the same temperature and duration. Diaminobenzidine solution was used to stain the sections. The staining reactions were observed carefully under a microscope (BX51; Olympus Corporation, Tokyo, Japan) and stopped with tap water. Finally, the sections were counterstained with hematoxylin. Negative controls were obtained by omission of the primary antibodies in all the IHC procedures.

The sections were observed by two independent pathologists. IHC staining was classified according to the percentage of cells with a positive score for staining. Firstly, the staining intensity was divided into four levels and scored as follows: 0, negative; 1, weak; 2, moderate; and 3, high. Secondly, the percentage of positive cells was divided into four levels and scored as follows: 0, 0% positive cells; 1, <30% positive cells; 2, 30–70% positive cells; and 3, >70% positive cells. Subsequently, the sum of the two scores for staining intensity and the percentage of positive cells was used as a final score for each sample. Samples were classified as negative (−) if the final score was 0, weak positive (+) if the final scores were 1–2, moderate positive (++) if the final scores were 3–4 and strong positive (+++) if the final scores were 5–6. Plexin-B3 demonstrated membrane and cytoplasm staining; however, no signal was observed in the negative controls.

### Statistical analysis

Comparisons between groups were statistically analyzed using the two-tailed Student’s t-test and the χ^2^ test. Statistical analyses were performed using SPSS 19.0 (IBM, Armonk, NY, USA) software for Windows, where P<0.05 was considered to indicate a statistically significant difference.

## Results

### mRNA expression levels of plexin-B3 in HCC

**Figure 1:**
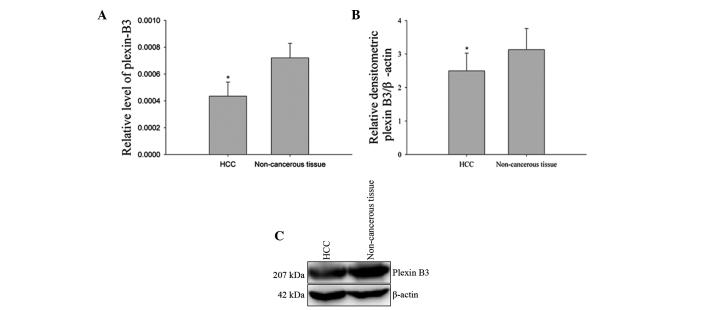
Expression of plexin-B3 in HCC. (A) Relative mRNA expression levels of plexin-B3 in the HCC samples and the corresponding adjacent non-cancerous tissue. Plexin-B3 expression was shown to decrease in the HCC samples when compared with the corresponding adjacent non-cancerous tissue. (B) Summary and (C) representative result from western blot analysis showing the protein expression levels of plexin-B3 in the 14 paired HCC samples and corresponding adjacent non-cancerous tissue. β-actin was used as a loading control. ^*^P<0.05, vs. non-cancerous tissue. HCC, hepatocellular carcinoma.

To examine the biological significance of plexin-B3 in HCC, 14 pairs of HCC samples and corresponding adjacent non-cancerous tissues were analyzed by quantitative PCR. The mRNA expression levels of plexin-B3 were found to be significantly decreased in 11 of the 14 (78.6%) HCC samples when compared with the corresponding adjacent non-cancerous tissue (P<0.05, two-tailed Student’s t-test; Fig. 1A). These results indicated that plexin-B3 may play a tumor suppressor role in hepatocarcinogenesis.

### Protein expression levels of plexin-B3 in HCC

Protein expression levels of plexin-B3 were analyzed in HCC samples and the corresponding adjacent non-cancerous tissue by western blot analysis. A representative result of western blot analysis for the expression of plexin-B3 is shown in Fig. 1. The protein expression levels of plexin-B3 were found to be downregulated in the HCC samples when compared with the corresponding adjacent non-cancerous tissue (P<0.05, two-tailed Student’s t-test; Fig. 1B and C), which was consistent with the results from the quantitative PCR analysis.

### Immunohistochemical characteristics

**Figure 2:**
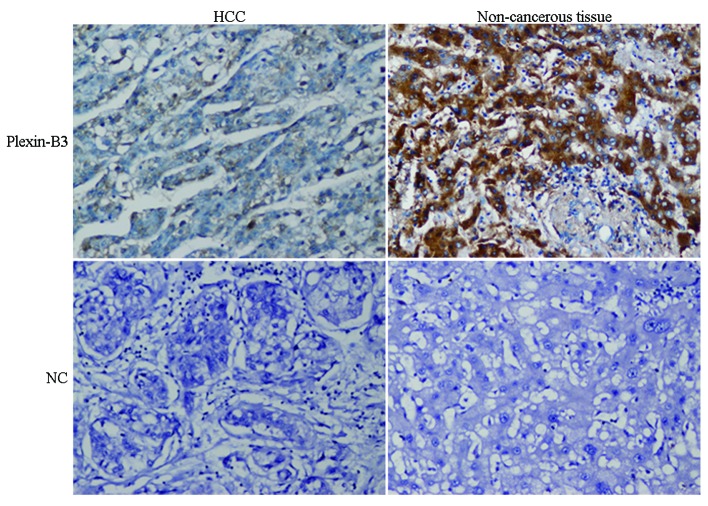
Expression of plexin-B3 in the HCC samples and the corresponding adjacent non-cancerous tissue was analyzed using immunohistochemistry (DAB staining, hematoxylin counterstain). More than half of all cases (52.4%) of plexin-B3 staining in HCC tissues was negative, whereas the majority of cases (70.2%) of plexin-B3 staining in the corresponding adjacent non-cancerous tissue was positive. Representative images are shown. The results of the NC are also shown (magnification, ×200). HCC, hepatocellular carcinoma; NC, negative control.

**Figure 3:**
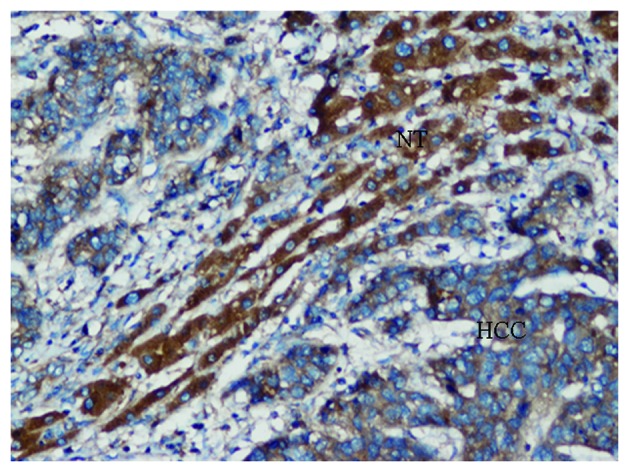
Staining of plexin-B3 in the corresponding adjacent non-cancerous tissue by immunohistochemistry analysis (DAB staining, hematoxylin counterstain). A representative image of hepatocytes exhibiting strong cytoplasmic staining in the non-cancerous tissue (magnification, ×200). HCC, hepatocellular carcinoma; NT, non-cancerous tissue.

Observations of the hematoxylin and eosin-stained sections revealed the HCC cells to be relatively homogenous when the necrotic, hemorrhagic and fibrotic components were excluded. Plexin-B3 expression was negative or low in the majority of HCC samples, with positive staining observed in the membrane and cytoplasm of the tumor cells in positive cases. Only 40 samples of the 84 cases exhibited positive expression, with a positive expression rate of 47.6%. In the corresponding adjacent non-cancerous tissue, membrane and cytoplasm staining of plexin-B3 were observed in hepatocyte cells. The majority of cases (70.2%; 59/84) revealed staining patterns with an intermediate or strong staining intensity, with a positive score for plexin-B3 expression in 84 HCC patients (Figs. 2 and 3). Statistical analysis indicated that plexin-B3 expression was significantly downregulated in the HCC samples when compared with the adjacent non-cancerous tissue (P<0.05, χ^2^ test; Table I).

In order to improve the understanding of the potential roles of plexin-B3 in HCC development and progression, associations between plexin-B3 expression and clinicopathological characteristics of HCC patients were analyzed. The results indicated that the loss of plexin-B3 expression was associated with the patient gender (P=0.01, χ^2^ test; Table II) and tumor size (P=0.001, χ^2^; Table II); however, there were no correlations with age, histology, tumor stage, tumor number, capsule invasion, microvascular invasion and liver cirrhosis (Table II).

## Discussion

Plexins are receptors for multiple classes of semaphorins that mediate the function of semaphorins, alone or in combination with neuropilins, including roles in cell repulsion, integrin function, cell migration and cell survival (6,10,24). In recent years, there has been increasing evidence indicating the important roles that plexins play in a variety of tumor initiation and progression processes (9,11). In the present study, the expression of plexin-B3 was analyzed in HCC samples and corresponding adjacent non-cancerous tissue using quantitative PCR and western blot analysis. The results revealed that the mRNA and protein expression levels of plexin-B3 were significantly downregulated in the HCC samples when compared with the corresponding adjacent non-cancerous tissue. There is a possible tendency for plexin-B3 to function as a putative tumor suppressor in HCC. The spatial distribution and expression levels of plexin-B3 were further confirmed by IHC staining. Plexin-B3 positive staining was observed in the membrane and cytoplasm of the tumor cells and hepatocytes. Plexin-B3 immunoreactivity in the HCC samples was significantly lower compared with the corresponding adjacent non-cancerous tissue, and the loss of plexin-B3 expression was found to correlate with the patient gender and tumor size. In addition, plexin-B3 expression levels in female HCC patients were significantly lower compared with those in male HCC patients, and the positive rate of plexin-B3 staining was significantly decreased in tumors of a large size (>5 cm in diameter) compared with tumors of a small size (≤5 cm in diameter). These results indicate that plexin-B3 may be used as a potential biological target for the diagnosis, progression and prognosis of HCC.

Semaphorins and their receptors, including plexins and neuropilins, are aberrantly expressed in human tumors, and can promote or inhibit cancer progression, with certain receptors exerting a dual role (11). Kantor *et al* (25) reported that Sema5A is a bifunctional guidance cue, which exerts both attractive and inhibitory effects on developing axons of the fasciculus retroflexus. The neuronal responses to Sema5A are regulated by heparin and chondroitin sulfate proteoglycans. Recently, several studies have reported that Sema5A has a dual effect on cell migration (26–28). Li and Lee found that Sema5A inhibited the Rac1 GTPase through stimulation of plexin-B3, which resulted in the inhibition of glioma cell migration and invasion (16). However, overexpression of Sema5A in pancreatic cancer has been shown to correlate with invasion, metastasis and increased endothelial cell proliferation (28,29), while overexpression of Sema5A and plexin-B3 are associated with the invasion and metastasis of gastric carcinoma (20). In the present study, in order to improve the understanding into the expression and role of plexin-B3 in HCC as a specific Sema5A receptor, a number of assays were performed. Plexin-B3 expression in HCC cases was shown to be downregulated, indicating that plexin-B3 may exert a suppressive effect on HCC tumors. Therefore, it is possible that the Sema5A/plexin-B3 signaling pathway may exert tumor promoting and suppressive effects depending on the type of malignancy.

In conclusion, to the best of our knowledge, the present study is the first to investigate the expression levels of plexin-B3 in HCC samples and the associations with clinicopathological data. The results indicated that plexin-B3 expression is downregulated in HCC, and the expression levels correlate with gender and tumor size. However, future studies are required to investigate the exact mechanisms underlying the role of plexin-B3 in the progression of HCC. Although the current results are not able to describe the full properties of plexin-B3 in HCC, the observations indicate that plexin-B3 plays an important role in the development and occurrence of HCC; thus, the receptor may be used as a predictive and therapeutic biomarker.

## Figures and Tables

**Table I:** Expression of plexin-B3 in 84 cases of HCC samples and adjacent non-cancerous tissue by IHC analysis.

		Plexin-B3 expression				
			
Tissue type	Cases (n)	(+++)	(++)	(+)	(−)	P-value
HCC samples	84	10	17	13	44	0.003
Adjacent non-cancerous tissue	84	12	20	27	25	

χ^2^ test was used to statistically analyze these data. HCC, hepatocellular carcinoma; IHC, immunohistochemistry.

**Table II:** Clinicopathological features of the HCC patients with positive and negative expression of plexin-B3.

		Plexin-B3 expression (n)		
			
Pathological features	Cases (n)	Positive	Negative	P-value
Gender				
Male	68	27	31	0.01
Female	16	3	13	
Age (years)				
>50	37	19	18	0.543
≤50	47	21	26	
Histology				
Well	12	9	3	0.121
Moderately	54	23	31	
Poor	18	8	10	
Tumor stage				
I+II	76	36	40	0.887
III+IV	8	4	4	
Node number (NI, 3)				
Single	70	31	39	0.232
Multiple	11	7	4	
Tumor size (cm; NI, 3)				
>5	30	7	23	0.001
≤5	51	31	20	
Capsule invasion				
Yes	41	19	22	0.819
No	43	21	22	
Microvascular invasion				
Yes	41	20	21	0.835
No	43	20	23	
Liver cirrhosis				
Yes	49	27	22	0.104
No	35	13	22	

χ^2^ test was used to statistically analyze these data. NI, no information; HCC, hepatocellular carcinoma.
